# Frequent post-operative monitoring of colorectal cancer using individualised ctDNA validated by multiregional molecular profiling

**DOI:** 10.1038/s41416-021-01266-4

**Published:** 2021-03-03

**Authors:** Mizunori Yaegashi, Takeshi Iwaya, Noriyuki Sasaki, Masashi Fujita, Zhenlin Ju, Doris Siwak, Tsuyoshi Hachiya, Kei Sato, Fumitaka Endo, Toshimoto Kimura, Koki Otsuka, Ryo Sugimoto, Tamotsu Sugai, Lance Liotta, Yiling Lu, Gordon B. Mills, Hidewaki Nakagawa, Satoshi S. Nishizuka

**Affiliations:** 1 Department of Surgery, Iwate Medical University School of Medicine, Iwate, Japan; 2 Department of Surgery, Iwate Prefectural Kuji Hospital, Iwate, Japan; 3 Division of Biomedical Research and Development, Iwate Medical University Institute for Biomedical Sciences, Iwate, Japan; 4 Laboratory for Cancer Genomics Genome Sequencing Analysis, RIKEN Center for Integrative Medical Sciences, Tokyo, Japan; 5 Department of Bioinformatics and Computational Biology, The University of Texas, MD Anderson Cancer Center, Houston, TX USA; 6 Department of Genomic Medicine, The University of Texas, MD Anderson Cancer Center, Houston, TX USA; 7 Division of Biomedical Information Analysis, Iwate Tohoku Medical Megabank Organization, Iwate Medical University, Iwate, Japan; 8 Department of Molecular Diagnostic Pathology, Iwate Medical University School of Medicine, Iwate, Japan; 9 Center for Applied Proteomics and Molecular Medicine, George Mason University, Manassas, VA USA; 10 Department of Cell, Development & Cancer Biology, Knight Cancer Institute Oregon Health & Science University, Portland, OR USA

**Keywords:** Rectal cancer, Cancer genomics, Colon cancer, Tumour biomarkers

## Abstract

**Background:**

Circulating tumour DNA (ctDNA) is known as a tumour-specific personalised biomarker, but the mutation-selection criteria from heterogeneous tumours remain a challenge.

**Methods:**

We conducted multiregional sequencing of 42 specimens from 14 colorectal tumours of 12 patients, including two double-cancer cases, to identify mutational heterogeneity to develop personalised ctDNA assays using 175 plasma samples.

**Results:**

“Founder” mutations, defined as a mutation that is present in all regions of the tumour in a binary manner (i.e., present or absent), were identified in 12/14 tumours. In contrast, “truncal” mutations, which are the first mutation that occurs prior to the divergence of branches in the phylogenetic tree using variant allele frequency (VAF) as continuous variables, were identified in 12/14 tumours. Two tumours without founder and truncal mutations were hypermutators. Most founder and truncal mutations exhibited higher VAFs than “non-founder” and “branch” mutations, resulting in a high chance to be detected in ctDNA. In post-operative long-term observation for 10/12 patients, early relapse prediction, treatment efficacy and non-relapse corroboration were achievable from frequent ctDNA monitoring.

**Conclusions:**

A single biopsy is sufficient to develop custom dPCR probes for monitoring tumour burden in most CRC patients. However, it may not be effective for those with hypermutated tumours.

## Background

Colorectal cancer (CRC) is the third most common cancer diagnosed worldwide with approximately 1,800,000 new cases and approximately 881,000 deaths in 2018.^1^ Post-operative relapse with distant metastasis (mCRC) represents the major cause of CRC-related deaths. Currently, computerised tomography (CT) scans and serum carcinoembryonic antigen (CEA) detection remain the gold standard to detect post-operative relapse and evaluate treatment response for metastases. Recently, randomised trials have not demonstrated significant survival benefit from the intensive follow-up, and the optimal surveillance strategy remains as yet unknown.^2–5^ Thus, to detect post-operative relapse in a timely manner and precisely evaluate therapeutic response, a quantitative patient tumour-specific marker for frequent monitoring of tumour burden dynamics in daily clinical practice is needed.

Circulating tumour DNA (ctDNA) represents an emerging tumour marker.^6^ The potential utility of ctDNA is based on the principle that somatic mutations are derived exclusively from cancer cells and thus may facilitate earlier detection of post-operative recurrence compared to conventional serum tumour markers and imaging approaches.^7^ In addition to recurrence monitoring, ctDNA can be used to select patients for molecular targeting drugs, particularly when biopsy samples cannot be obtained.^8^ A major challenge to the utility of ctDNA as an individualised tumour marker is the need to account for mutational tumour heterogeneity both between patients and within patients as a consequence of tumour evolution and treatment.^9–12^ Although DNA fragments are released from heterogeneous tumour cells, whether mutational heterogeneity and phylogenetic characteristics based on multiregional sequence data across a tumour are fully reflected in ctDNA remains unclear.^13^

Another challenge of implementing ctDNA monitoring in daily practice is the identification of a sensitive and practical detection system for frequent monitoring of the very low variant allele frequencies (VAFs) present in blood. The detection limit of VAFs by next-generation sequencing (NGS) is generally >1%.^7^ Indeed, the majority of ctDNA VAF is <1%, even in recurrent cases.^14^ In the present study, we implemented a highly specific and personalised digital PCR (dPCR) primer/probe approach using Hypercool Primer & Probe (HPP) technology to robustly detect mutations identified in resected tumour specimens from that patient.^15,16^ In our system, dPCR with HPP technology has a 0.01% VAF detection limit as well as >95% successful reaction rate.^17^ The high dPCR success rate facilitates a cost-effective and frequent patient-specific tumour burden monitoring approach in daily practice that can facilitate (a) early relapse prediction, (b) treatment efficacy evaluation and (c) non-relapse corroboration. Here, we present the evaluation of genomic characteristics of post-operative Stage III/IV CRC along with the results for frequent ctDNA monitoring conducted over approximately 1000 follow-up days.

## Methods

### Study design

The subjects enrolled in this study were analysed as part of an observational study. All enrolled patients had intention-to-treat for pathological Stage III or more advanced CRC and were treated at the Department of Surgery, Iwate Medical University School of Medicine between March 2016 and December 2016 (HGH27-29). In principle, all patients received treatment for advanced CRC according to the Japanese Society for Cancer of the Colon and Rectum guidelines.^18^ No specific intervention and specifically scheduled clinical examinations for this particular study were allowed. Pre- and post-operative peripheral blood samples were obtained in addition to routine clinical laboratory examinations.

### Sampling and preservation

All primary tumour tissues were post-operatively acquired prior to formalin fixation. Based on a macroscopic diagnosis of resected tumours, samples were taken from three regions in the tumour that was spaced at least five millimetres apart. Each sample was divided into two portions from which DNA was extracted and cell lysates were prepared before storage at −80 °C. The first whole-blood sample was collected into a BD Vacutainer CPT blood collection tube (Becton, Dickinson and Company, East Rutherford, NJ). The whole-blood sample was separated into plasma and peripheral blood mononuclear cells (PBMCs) that were used to analyse germline DNA. Within 2 h of collection, both plasma and PBMC phases were further centrifuged separately at 1800×*g* at room temperature to remove other components. The follow-up blood samples were acquired “frequently” (e.g., every 1–3 months) such that collection was carried out simultaneously with routine clinical-pathway laboratory examinations in 2 × 10-ml volumes in two Cell-Free DNA Streck BCT blood collection tubes (Streck, Omaha, NE) at room temperature. Within 5 days of blood withdrawal into a BCT tube stored at room temperature, the blood sample was centrifuged at 1800×*g* for 20 min at room temperature to separate plasma and red blood cells. The plasma phase was transferred to another tube that was centrifuged at 1800×*g* for 20 min at room temperature to remove cellular debris. The isolated plasma phase was stored at −80 °C.

### DNA extraction

The genomic DNA was extracted using a QIAamp DNA Mini Kit for tumour tissue and PBMCs, and a QIAamp Circulating Nucleic Acid Kit for plasma (Qiagen, Germany). The extracted DNA in solution was transferred to a 0.5-ml tube and stored at −30 °C until analysis. The quantity of extracted DNA was measured using a Qubit12.0 dsDNA high-sensitivity assay kit (Life Technologies, Carlsbad, CA).

### Panel sequence

For each patient, genomic DNA was extracted from three regions of the primary tumour and from PBMCs. NGS libraries were prepared using ClearSeq Comprehensive Cancer Kits according to the manufacturer’s instructions (Agilent Technologies, Inc., USA). The ClearSeq Comprehensive Cancer Panel targets 151 disease-associated genes and was analysed using an Illumina Hiseq 2000 (enrichment system) (Supplementary Table 1, Illumina, Inc., San Diego, CA). Qualification of variant calling was performed by adaptor-trimming of reads using Cutadapt (http://code.google.com/p/cutadapt/) and mapping to GRCh37 using Burrows-Wheeler Aligner.^19^ PCR duplicates were removed using Picard (https://broadinstitute.github.io/picard/). Low-quality reads were discarded when they had a mapping quality less than 20, three or more mismatched bases or two or more INDELs. Paired-end reads were also filtered out when they were mapped onto different chromosomes, mapped in improper directions or their insertion length exceeded the mean ± 3 standard deviations. SNVs and INDELs were called using VarScan2^20^ with a minimum read depth of 20, a minimum variant allele frequency of 5%, minimum supporting reads of four and a p-value threshold of 0.05. The variants were annotated using Ensembl VEP. Copy number (CN) analysis was performed using VarScan2 and DNAcopy.^21^

### Copy number variation

Copy number variation (CNV) was calculated using ONCOCNV obtained via GitHub,^22^ with BAM files as input. Read counts in tumour BAM files were normalised, corrected for colon cancer content and the CNV was detected by comparison with the baseline copy number. The baseline copy number (CN) was defined based on PBMC BAM files and was subsequently used for CNV calculation for all multiregional samples. CN segmentation was performed using the DNAcopy package of R/Bioconductor. ONCOCNV was run on the SHIROKANE supercomputer at the University of Tokyo Institute of Medical Science.

### Phylogenetic tree

Phylogenetic trees were constructed using a modified version of Canopy (version 1.3.0), an open-source R package (https://cran.r-project.org/web/packages/Canopy/).^13^ A list of somatic SNV/INDELs was used as input. Based on preliminary simulations of evolutionary trees, SNV data were prioritised for the simulation in the present study in which CNV data were limited to those from the panel sequencing. Clustering of SNV/INDELs was performed during pre-processing to accelerate simulation convergence. Markov chain Monte Carlo simulations were run 10 times, and the maximum simulation length was 100,000 steps. The convergence of simulations was confirmed by visually inspecting the time course of log-likelihood and acceptance rate in all cases. Data from the burn-in phase were discarded.

### dPCR

Mutations in *KRAS* and *BRAF* and *PIK3CA* were analysed using a commercially available primer/probe kit for dPCR (Thermo Fisher Scientific). Primer/probe sets for SNVs were designed and synthesised using Hypercool^TM^ Primer & Probe technology (Nihon Gene Research Laboratories, Inc., Sendai, Japan) wherein forward and reverse primers and a hydrolysis probe were designed that would produce an amplicon of ~70 bp. Several adenine or cytosine bases in these primers/probes were replaced with “2-amino-dA(2 aA)” and “5-Methyl-dC(5mC)”, respectively, to ensure a high Tm value despite the short amplicon length. Primer/probe sets originally designed using Hypercool^TM^ Primer & Probe technology were validated for use with dPCR and DNA from primary tumours (Nihon Gene Research Laboratories, Sendai, Japan). The QuantStudio 3D Digital PCR System (Thermo Fisher Scientific) was used for PCR and counting of absolute mutation fragments. The maximum input of plasma cfDNA ranged from 0.2–8.5 µl per dPCR assay. VAF was calculated when at least two mutant-type signal dots (FAM) at one time point or more than one mutant-type signal dot at consecutive time points existed in the presence of wild-type signal dots (VIC) using the formula:

$${\mathrm{VAF}}\left( {\mathrm{\% }} \right) = \frac{{{\mathrm{Mutant}} - {\mathrm{type}}\;{\mathrm{dots}}\,\left( {{\mathrm{FAM}}} \right)}}{{{\mathrm{Mutant}} - {\mathrm{type}}\;{\mathrm{dots}}\,\left( {{\mathrm{FAM}}} \right) + {\mathrm{Wild}} - {\mathrm{type}}\;{\mathrm{dots}}\,\left( {{\mathrm{VIC}}} \right)}} \times 100\left( \% \right)$$

### Statistical analysis

Both parametric and nonparametric tests for group comparison were performed, and included Student’s *t* test, Wilcoxon signed-rank test, Mann–Whitney *U* test for continuous variables, chi-square test and Fisher’s exact test for categorical variables and Pearson’s or Spearman’s correlation coefficient for two variables. All statistical analyses were performed using JMP software version 14.0 (SAS Institute, Inc., Cary, NC, USA). A probability (*P*) value of < 0.05 was considered statistically significant.

## Results

### Patient characteristics

In our prospective observational study, 44 clinical Stage II–IV CRC patients with disease that was considered to be resectable at the time of diagnosis were registered between March 2016 and December 2016. Two patients were excluded due to unresectable lesions and pathological Stage I disease. The other 42 patients that were confirmed to have pathological Stage II–IV CRC underwent primary tumour resection. For the present study, 12 patients were enrolled based on the following criteria: (a) availability of three samples collected from a primary tumour that was at least pathological Stage III, and (b) confirmation of tumour cellularity >40% in all specimens (Supplementary Fig. 1 and Supplementary Table 2). The final number of tumour regions sequenced was 42, which were taken from 14 tumours obtained from 12 patients. Among these patients, two patients had two separate colorectal tumours.

### Multiregional sequence of primary tumours by NGS

**Fig. 1:**
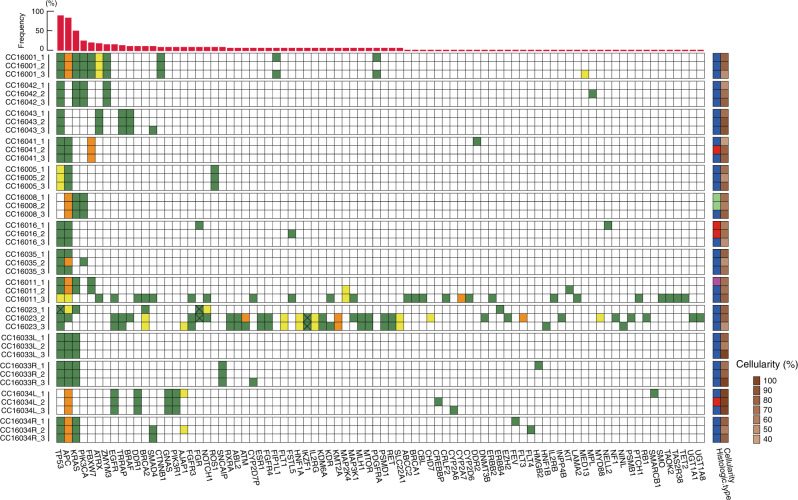
Multiregional sequencing of primary tumours. The horizontal axis shows genes that were mutated in at least one sample and the vertical axis lists case number and sample region. Green and yellow squares indicate a single-nucleotide variant (SNV) and insertion/deletion variant (INDEL), respectively. Orange squares indicate the presence of both SNV and INDEL. The same colour square for a gene in identical tumours indicates the presence of the same genetic mutation; however, the x-mark in the same colour squares means that these mutational locations are different from each other. These mutations show non-synonymous variants. Light green, red, blue and magenta squares in the right-hand column indicate papillary, well-differentiated, moderately differentiated and poorly differentiated adenocarcinomas, respectively. The red bar graph at the top indicates the frequencies of mutations in 42 sample regions.

Multiregional sequences were performed for 42 specimens from 14 colorectal primary tumours of 12 patients. Sequence analysis was performed using the ClearSeq Comprehensive Cancer panel that targets 151 disease-associated genes (Supplementary Table 1). A total of 157 mutations from 84 genes were identified in the 14 primary tumours from 12 patients, including two double-cancer cases (Fig. 1). The average number of mutations per tumour was 12.1 (range: 3–55), whereas the average in a single region was 7.2 (range: 2–43). For this study, a founder mutation was defined as a mutation that is present in three regions of the tumour in a binary manner (i.e., present or absent); 12/14 (85.7%) tumours had founder mutations. Patients CC16011 and CC16023 had no founder mutations but did have 40 and 55 non-founder mutations, respectively. In the 42 samples examined, 156 founder mutations (45 unique point mutations) and 147 non-founder mutations (113 unique point mutations) were identified. The average number of founder and non-founder mutations per tumour was 3.7 (range: 0–9) and 8.4 (range: 0–55), respectively. The median VAF (%) of founder mutations was significantly higher than that of non-founder mutations (30.0 [IQR, interquartile range: 22.9–41.4] vs. 22.4 (IQR: 12.1–38.8), *P* < 0.0001, Supplementary Table 3).

### Phylogenetic trees for primary tumours

**Fig. 2:**
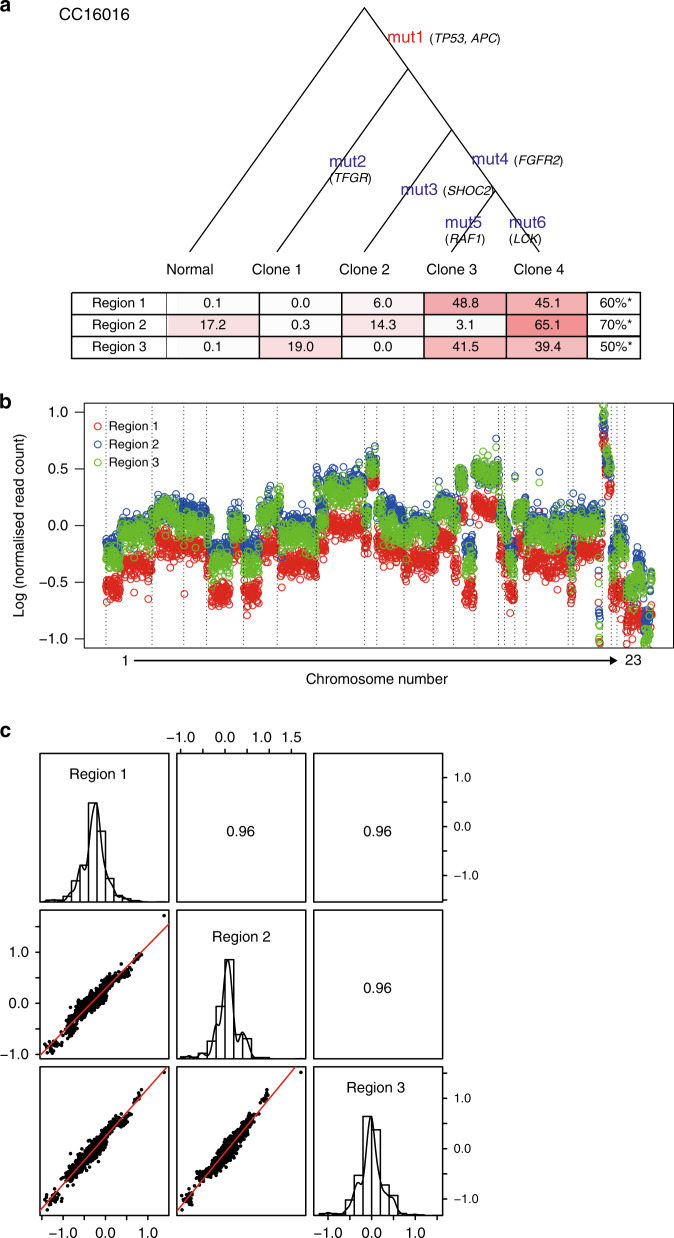
Intra-tumour genetic heterogeneity represented by the phylogenetic tree and CNV. **a** Letters in red and blue indicate truncal and branch mutations, respectively. The tables at the bottom of the phylogenic tree show simulated proportions (%) of each clone for three sample regions of the primary tumour. The set of mutations per sample is provided in Data file S1. **b** Colour dot representation of copy number across chromosomes in three regions of a tumour taken from CC16016. **c** Pearson’s correlation coefficients for all possible CNV combinations between three sample regions. Scatterplots are of two CNs of a given pair, whereas histograms show the frequency of CN distribution of a region. *Cellularity as the pathological content (%) was indicated at each region for the primary tumour.

Phylogenetic trees were generated to simulate cancer clonal composition and chronological evolution based on a multiregional mutation profile.^13^ A simulation for CC16016 yielded multiple clonal compositions in an individual region and also showed the relative timing of the introduction of new mutations (Fig. 2a). To understand chronological evolution, here, a “truncal” mutation was defined as the first mutation that occurred prior to the divergence of branches in the phylogenetic tree. The median VAF (%) of truncal mutations (71.5 (IQR: 55.7–80.5)) of CC16001 was significantly higher than that for branch mutations (26.7 (IQR: 20.1–37.1)) (*P* < 0.0001). The average number of truncal mutations per tumour was 1.4 (range 0–3), whereas the average for “branch” mutations was 72.6 (range 13–464). As predicted, in most tumours, the median VAF (%) of truncal mutations (48.6 (IQR: 37.1–60.9)) was significantly higher than that for branch mutations (21.2 (IQR: 9.4–30.7)) (*P* < 0.0001, Supplementary Table 4). Analyses for all tumour samples are shown in Fig. 2a and Supplementary Fig. 2.

### Founder and truncal mutations

The founder mutation is defined in a binary manner (i.e., presence or absence), whereas the truncal mutation is a result of statistical simulation in a quantitative manner using VAF per mutation per region. Among 14 tumours, 12 tumours had at least one founder mutation from a total of 52 founder mutations, whereas 12 tumours had at least one truncal mutation from a total of 19 truncal mutations. Hypermutators were defined as those with >10 mutations/Mb, CC16011 and CC16023 hypermutators, did not have founder mutations identified by NGS (Fig. 1) or truncal mutations by the Canopy simulation (Supplementary Fig. 2). Hence, we excluded these hypermutators from enumeration of founder/truncal mutations because it cannot be assumed that the tumour developed from a single transformed cell. Excluding hypermutators, 52 founder and 19 truncal mutations were identified among all 248 types of mutations found in 12 tumours. In addition, 18 founder mutations (18/52, 34.6%) were also truncal mutations. Importantly, the great majority (18/19, 94.7%) of truncal mutations were founder mutations.

### Intra-tumour copy number variation

Since a sequencing panel was used to identify single-nucleotide variations (SNVs) and insertion–deletion mutations (INDELs), the ability to assess copy number variations (CNVs) was less comprehensive than a whole-genome sequence. In fact, using sequencing results from the current cancer panel, the average number and size of detected CNVs were 16.6 events and 19.3 Mb, respectively. We used the ONCOCNV algorithm to characterise large copy number changes from gene panel sequencing.^22^ CNVs occurring in two arbitrary loci indicated strong correlations (r > 0.9) (Fig. 2b, c). In 42 possible combinations from three regions of 14 tumours, the median within-patient correlation coefficient was 0.86 (IQR: 0.70–0.92) (Supplementary Fig. 2). Based on the normalised CNV across 42 samples, 184 and 140 genetic regions were identified as having gain and loss of copy number, respectively (Supplementary Fig. 3). Overall, the high correlation among sample regions and notable CNVs suggests that CNVs, including some that have potentially critical functions, likely occurred at a relatively early stage during tumour development.

### Validation of mutations using dPCR

A set of 34 unique mutations for all tumours was selected to monitor the tumour burden. dPCR was used to confirm the concordance of VAFs between dPCR and NGS. Among the available DNA samples, tumour genetic heterogeneity was evaluable by dPCR in 121/123 (41 mutations × 3 regions) specimens. Among the 121 mutations from multi-region samples, 103 were identified by NGS, whereas the remainder were not, leaving the possibility that a mutation was not detected due to the low VAFs. Of the 18 mutations from one of the multi-regions that had not been detected by NGS, which were thought to be concordant with the rest of the two regions, 10 (55.6%) were detected by dPCR (Supplementary Data File 1). Of these mutations, half showed <1% VAF, likely accounting for the discordance. The overall concordance in mutation detection in a binary manner (i.e., presence or absence) between NGS and dPCR was 91.7% (111/121 mutations, Supplementary Data File 2). The VAFs measured by NGS and dPCR showed a good correlation, particularly in the high (>1%) range (*r* = 0.79, Supplementary Fig. 4).

### ctDNA in preoperative plasma samples

The average amount of preoperative cell-free DNA (cfDNA) in 1 mL plasma was 14.3 ng (range 7.2–34.2). Preoperative tumour-specific mutations were detected as ctDNA in 9/12 patients (75.0%) using dPCR. The average VAF of ctDNA for all disease stages assessed by dPCR was 1.02% ± 5.1 (±2 SD). By stage, the average VAF of preoperative ctDNA for Stage III and Stage IV A–B was 0.60% ± 1.32 (±2 SD) and 3.67% ± 10.12 (±2 SD), respectively. The detection rates for ctDNA of founder and non-founder mutations were 67.9% (19/28 mutations) and 61.5% (8/13 mutations), respectively; those of truncal and branch mutations were 75.0% (9/12 mutations) and 62.1% (18/29), respectively (Supplementary Fig. 5 and Supplementary Table 5). As expected, the detection rate for ctDNA of founder mutations was higher than that of non-founder mutations, and the detection rates as ctDNA of truncal mutations were higher than those of branch mutations. Among primary tumours with >10% VAF mutations, as well as >100 read counts by NGS, 74.0% (77/104) of detected ctDNA were derived from either founder or truncal mutations. Of note, the correlation coefficients between cellularity (%) and VAFs of preoperative ctDNA, or between cell-free DNA (cfDNA) concentration and VAFs of preoperative ctDNA were low (*r* = 0.19, *n* = 123; *r* = −0.14, *n* = 41, respectively), suggesting that the sample tumour cellularity or cfDNA concentration did not dominantly impact ctDNA VAFs. Overall, high VAF mutations seemed to be a reasonable surrogate of either founder or truncal mutations.

### ctDNA monitoring by dPCR over time

In contrast to NGS, dPCR offers several advantages, including high sensitivity, rapid turn-around time and low cost. These factors allow the frequent monitoring of individual tumour-specific mutations. We propose that ctDNA monitoring as a tumour marker could contribute to (a) early relapse detection, (b) treatment efficacy evaluation and (c) non-relapse corroboration. As examples, we consider the following cases. In this study, day 0 indicates the day post surgery for the primary tumour.

**Fig. 3:**
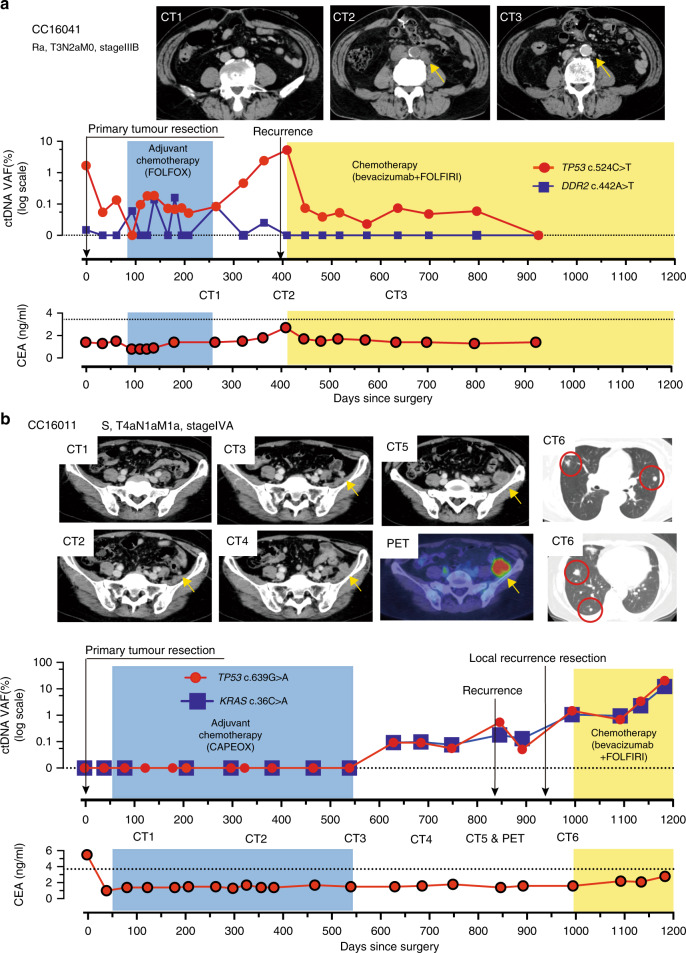
Early relapse prediction by ctDNA monitoring recurrent cases. **a** An aortic lymph node recurrence was noted by CT2 on day 409 after primary tumour resection. As noted in CT3, the size of the aortic lymph node recurrence was reduced on day 622. **b** Levels of ctDNA were undetectable up to day 539 during preoperative and adjuvant chemotherapy. Yellow arrowheads in CT images indicate a suspected local recurrence lesion in the pelvic peritoneum; this diagnosis was confirmed on day 846 (CT5 and PET). Continuous detection of ctDNA was possible for seven months before the recurrence was diagnosed by CT scan. Multiple lung metastases were diagnosed at day 980, after surgery for recurrence in the peritoneum (CT6). The patient began receiving chemotherapy on day 1015, at which point the ctDNA declined temporarily before re-increasing through day 1200. PET, positron emission computerised tomography.

Patient CC16041 had Stage III rectal cancer and underwent surgery with curative intent. The tumour from this patient carried a founder mutation for *TP53* (c.524C>T) and a non-founder mutation for *DDR2* (c.442A>T), which were both selected to assess the efficacy of ctDNA monitoring (Fig. 3a). Preoperative ctDNA for both *TP53* and *DDR2* decreased immediately after surgery, with decreases in *TP53* ctDNA levels, which had the highest VAF, being more pronounced than those for *DDR2*. Although the levels of both mutated ctDNAs fluctuated during post-operative adjuvant chemotherapy (FOLFOX), they both remained low with levels around 0.1% or lower. Strikingly, *TP53* ctDNA showed a subsequent increase after completion of adjuvant chemotherapy (FOLFOX). The peak *TP53* ctDNA level occurred on day 409 on which a follow-up CT identified a recurrent lesion at a para-aortic lymph node (Fig. 3a, CT2). Importantly, the increase in *TP53* ctDNA preceded imaging detection of recurrence by 90 days. Subsequent bevacizumab + FOLFIRI therapy resulted in a decrease in the size of the recurrent lesion on day 622 as well as a decrease in *TP53* ctDNA (Fig. 3a, CT3). For this patient, the concomitant serum tumour marker CEA and DDR2 ctDNA did not reach “positive” levels during the entire treatment course. This suggests that the clone(s) that resulted in the recurrence contained the founder *TP53* mutation in a large fraction, but not the non-founder *DDR2* mutation.

Patient CC16011 exhibited a different pattern. ctDNA was not detectable pre-surgery and remained undetectable for >500 days after surgery. ctDNA levels became detectable at day 629 and remained elevated despite surgery to remove a recurrent tumour and subsequent chemotherapy (bevacizumab + FOLFIRI). Importantly, however, the increase in ctDNA preceded to local recurrence detected by CT on day 846, ~300 days after adjuvant chemotherapy (CAPOX) ended (Fig. 3b). Once again, ctDNA increases also preceded increases in CEA. These observations suggest that individual tumour-specific ctDNA monitoring can provide information on recurrence for patients who underwent tumour resection with curative intent.

**Fig. 4:**
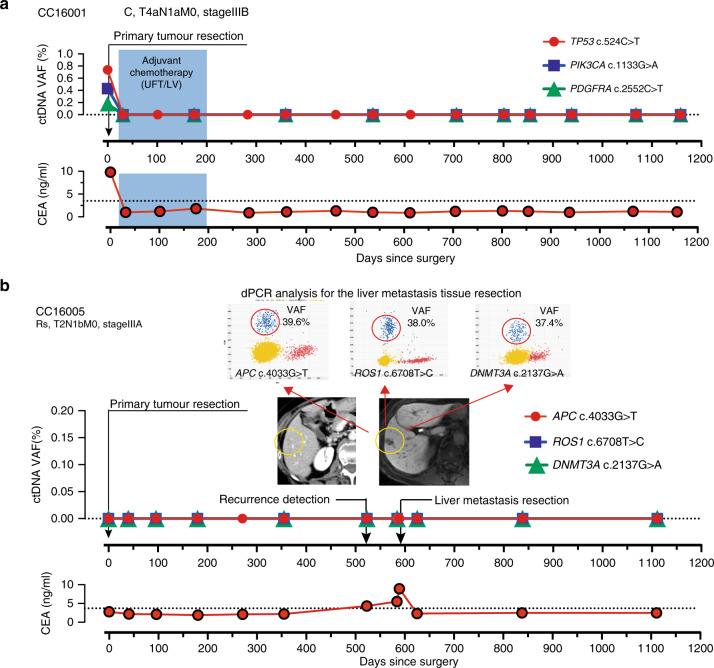
Undetected ctDNA monitoring by dPCR. **a** A case with no recurrence after primary colorectal cancer resection. **b** A patient was diagnosed with a single liver metastasis 1.5 years after the first operation. Three mutations in ctDNA were not detectable pre-operatively but were detected by dPCR in resected metastatic tissue.

Patient CC16001 demonstrated that ctDNA for three mutations was detectable in preoperative plasma (Fig. 4a). Post-operatively, ctDNA remained undetectable for nearly 1200 days. CEA levels for this patient dropped to standard levels after primary tumour resection. The estimated 3-year recurrence risk for CC16001 according to the final disease stage was ~25%.^23^ The continuous undetectable levels of ctDNA suggest that this patient was at low risk for recurrence, which was confirmed by a lack of recurrence for 1000 days. In the present case series, six additional patients (CC16008, CC16016, CC16023, CC16034, CC16042 and CC16043) with >Stage IIIA had a similar pattern to patient CC16001, whereby the levels of ctDNA that were detectable pre-therapy immediately decreased after tumour resection and remained undetectable for approximately 1000 days (Supplementary Fig. 6).

Patient CC16005 demonstrated another pattern. Despite concordance of mutations detected in the primary tumour and in a recurrent metastatic liver lesion, ctDNA was not detected throughout the patient’s course of management (Fig. 4b). Patient CC16035 had also undetectable ctDNA levels at pretreatment and throughout the treatment period with three mutations (Supplementary Fig. 7). While CC16035 did not recur, patient CC16005 developed a metastatic lesion that was not accompanied by an increase in ctDNA levels.

**Fig. 5:**
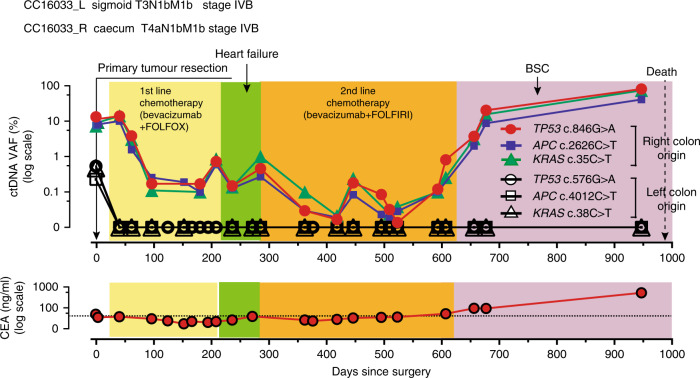
Longitudinal ctDNA monitoring in a patient with multiple cancer. This patient had two tumours in the caecum and sigmoid colon. He then had synchronous metastases of the liver and lung. A set of three mutations identified from the right colon tumour was detected as ctDNA after resection of both primary tumours and reflected the effect of chemotherapy. Another set of three mutations identified from the left colon tumour was only detected in preoperative plasma as ctDNA and subsequently remained undetectable. PD progressive disease, SD stable disease, BSC best supportive care.

Finally, patient CC16033 demonstrated another interesting pattern. CC16033 had the synchronous double- right (i.e., caecum) and left (i.e., sigmoid) colon tumours concomitant with metastases to the lung and liver (Supplementary Fig. 8a). Local resection of both primary tumours was performed, followed by chemotherapy. Metastatic lesions of the lung and the liver showed marginal responses to the two lines of chemotherapy (Supplementary Fig. 8b). Interestingly, both tumours had founder mutations of the same gene set (i.e., *TP53*, *APC* and *KRAS*) but the mutation positions were all different. Following primary tumour resection, ctDNA levels of one set of founder mutations identified from the left colon tumour decreased and remained undetectable (Fig. 5). However, ctDNA levels of another set of three founder mutations from the other (i.e., right) lesion did not decrease after surgery but did decrease during chemotherapy to near the detection limit (0.1%) of VAF. The ctDNA levels of the three founder mutations from the right tumour remained low until ~600 days post surgery when they began to increase and remained high until death. Importantly, the dynamics of two sets of three founder mutation levels remained concordant throughout the course of therapy, suggesting that they were from the same clone.

**Fig. 6:**
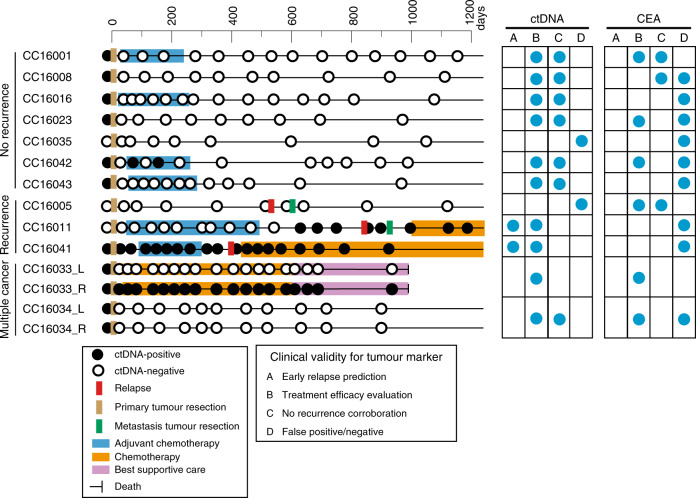
Longitudinal ctDNA status and individual clinical validities. ctDNA status and clinical information are illustrated on the horizontal lines by case. Blue circles in the right grid indicate representative clinical validities of ctDNA and CEA. **a** Early relapse prediction, **b** treatment efficacy evaluation, **c** non-relapse corroboration and **d** false positive/negative.

Our present series allowed us to estimate the clinical validity of ctDNA^24^ in terms of the above-mentioned categories (i.e., early relapse prediction, treatment efficacy evaluation and non-relapse corroboration). Overall, for 10 of the 12 patients analysed, there was information available in the personalised ctDNA analysis that could result in patient benefit with good contrast to conventional serum tumour marker, CEA (Fig. 6).

## Discussion

The ultimate clinical utility of tumour markers is their ability to provide unique information that can alter patient management leading to improved survival.^24^ Therefore, subjects who are most likely to benefit from tumour marker data are those with an intention-to-treat approach designed to reduce tumour burden and increase the survival rate.^25^ In a search for clinically useful tumour markers for patients with advanced-stage CRC, we examined ctDNA in terms of tumour heterogeneity, and the concordance to clinical events. Unique and practical information for clinical validity can be obtained from ctDNA including (a) early relapse detection, (b) treatment efficacy evaluation and (c) non-relapse corroboration. Our results suggest that the presence of high VAF mutations in a tumour can be a practical surrogate for founder mutations that are likely to be detected as ctDNA.^26^

To establish ctDNA as a clinically useful marker, exhaustive biological investigations are needed. In contrast to studies that focused on tumour phylogeny, here we assessed the genetic heterogeneity of tumours using a simple, minimum-number (i.e., three) multi-region sequencing, which was chosen to represent a practical number for standard clinical practice. Tumour-specific mutations for each tumour were selected from mutations identified from three regions. The high VAF mutations selected as tumour-specific mutations were more reflected in ctDNA monitoring using dPCR. The objective of the multi-region sequencing was to identify founder and truncal mutations and to consider intra-tumour heterogeneity. While a “founder” mutation is defined in a binary manner (i.e., presence or absence), “truncal” mutations take VAF (i.e., continuous variables) into account. We demonstrated that dPCR was able to detect the presence of mutations that had not been identified by NGS, which resulted in more founder mutations than expected. This observation may suggest, under the binary distinction, that if genetic heterogeneity is assessed with techniques whose quantitative detection limit is less than 0.1%, then the prevalence of the genetic heterogeneity may be less than previous studies exhibiting a lower depth of analysis suggested.^9,27,28^ In addition, we verified that the high VAF mutations were likely to be truncal mutations using Canopy, and founder mutations had higher VAF than non-founder mutations. Therefore, it is reasonable to use either founder or truncal mutations at the NGS sensitivity level for ctDNA monitoring, since these mutations should have a higher chance to be released from anywhere within the tumour. However, in patients with hypermutated tumours, mutation selection may require different criteria.

In daily practice, however, it is impractical to run multi-region sequencing in every single patient. The present results suggest that a high VAF may be a good surrogate of both founder and truncal mutations. Although multiregional sequencing and phylogenetic tree simulation^26^ provided a solid justification in terms of selecting mutations suitable for ctDNA monitoring, we suggest that high VAF mutations in a tumour from a single region may be sufficient for sequencing in daily clinical practice. In fact, using high VAF mutations from primary tumours for ctDNA monitoring, we showed that ctDNA provided a 3–6-month lead time compared to conventional imaging examinations for early relapse prediction. Moreover, in daily practice, both treatment efficacy and non-relapse corroboration could be assessed with greater frequency, which is likely to extend the lead time in case treatment action is needed. For those that did not exhibit positive preoperative ctDNA (CC16005 and CC16035), we suspect that the negative ctDNA, despite high tumour burden, was likely due to (i) technical difficulties in capturing founder/truncal mutations as ctDNA, (ii) lack of identification of good mutations from genetically heterogeneous tumours and (iii) primary tumours releasing very little ctDNA.

There are several limitations to the present study: (i) the number of enrolled patients (*n* = 12) was relatively small, (ii) the number of regions sampled (three per tumour) may not have allowed estimation of tumour-wide clonal heterogeneity, (iii) the number of mutations monitored that were specific to each patient was limited and was not designed to assess clonal changes and (iv) the survey period (median 965 days) may not be sufficiently long to evaluate late recurrence. Nevertheless, the ctDNA results provide clues for earlier detection of post-treatment relapse than the currently used CT scan. In addition, the cost of ctDNA monitoring is less expensive and lower risk than intensive CT scans during post treatment.

In summary, dPCR allows frequent and rapid assays with at least a 10-fold lower detection limit compared to NGS.^7^ A limited set of personalised mutation targets was sufficient to monitor tumour burden. Tumour genetic heterogeneity does not appear to represent a major obstacle for ctDNA monitoring if an appropriate high VAF somatic mutation is selected from a single biopsy. In patients with hypermutated tumours, however, further studies are needed to fully understand how ctDNA reflects tumour burden. Further interventional prospective studies are needed to confirm that ctDNA monitoring provides an effective approach for extending patient survival.

## Supplementary information

Supplemental Material clean

Supplementary Date file 1

Supplementary Date file 2

## Data Availability

Underlying data are available here: Gene sequencing panel: NBDC Human Database (https://humandbs.biosciencedbc.jp/en/hum0232-v1) Submission: JGA00000000284 Study: JGAS00000000243 Dataset: JGAD00000000343 RPPA data: The University of Texas MD Anderson Cancer Center RPPA data repository URL: https://tcpaportal.org Accession ID: TCPA00000006-1.
