# Supplementary material for: Frequent post-operative monitoring of colorectal cancer using individualised ctDNA validated by multiregional molecular profiling
Source: Br J Cancer. 2021 Mar 3;124(9):1556–65. doi: 10.1038/s41416-021-01266-4 (PMC8076308; doi:10.1038/s41416-021-01266-4)
Supplement: Supplementary file 1 — Supplemental Material clean [file 41416_2021_1266_MOESM1_ESM.docx]

**SUPPLEMENTARY MATERIALS**

**Supplementary Figure 1**. **Flow chart of HGH28-15 study**

**Supplementary Figure 2**. **Intra-tumor genetic heterogeneity represented by phylogenetic tree and CNV**

**Supplementary Figure 3**. **Copy number variations (CNVs) detected in each sample**

**Supplementary Figure 4**. **Correlation of VAF between NGS and dPCR in primary tumor**

**Supplementary Figure 5**. **VAFs of ctDNAs by founder and non-founder mutation**

**Supplementary Figure 6**. **No recurrence corroboration by ctDNA monitoring**

**Supplementary Figure 7**. **A case having undetectable ctDNA throughout the clinical course**

**Supplementary Figure 8. Information of a patient with multiple cancer and synchronous metastases**

**Supplementary Table 1**. **Targeted disease-associated genes in cancer panel (n=151)**

**Supplementary Table 2**. **Patient information**

**Supplementary Table 3**. **Mutations in primary tumors**

**Supplementary Table 4**. **Comparison between trunk and branch mutations**

**Supplementary Table 5**. **Information for tumor-specific mutations in each primary tumor**

**Supplementary Data file 1**. **Information for mutations in the phylogenetic tree for each case**

**Supplementary Data file 2**. **Mutations detected by dPCR in the regions examined by NGS**


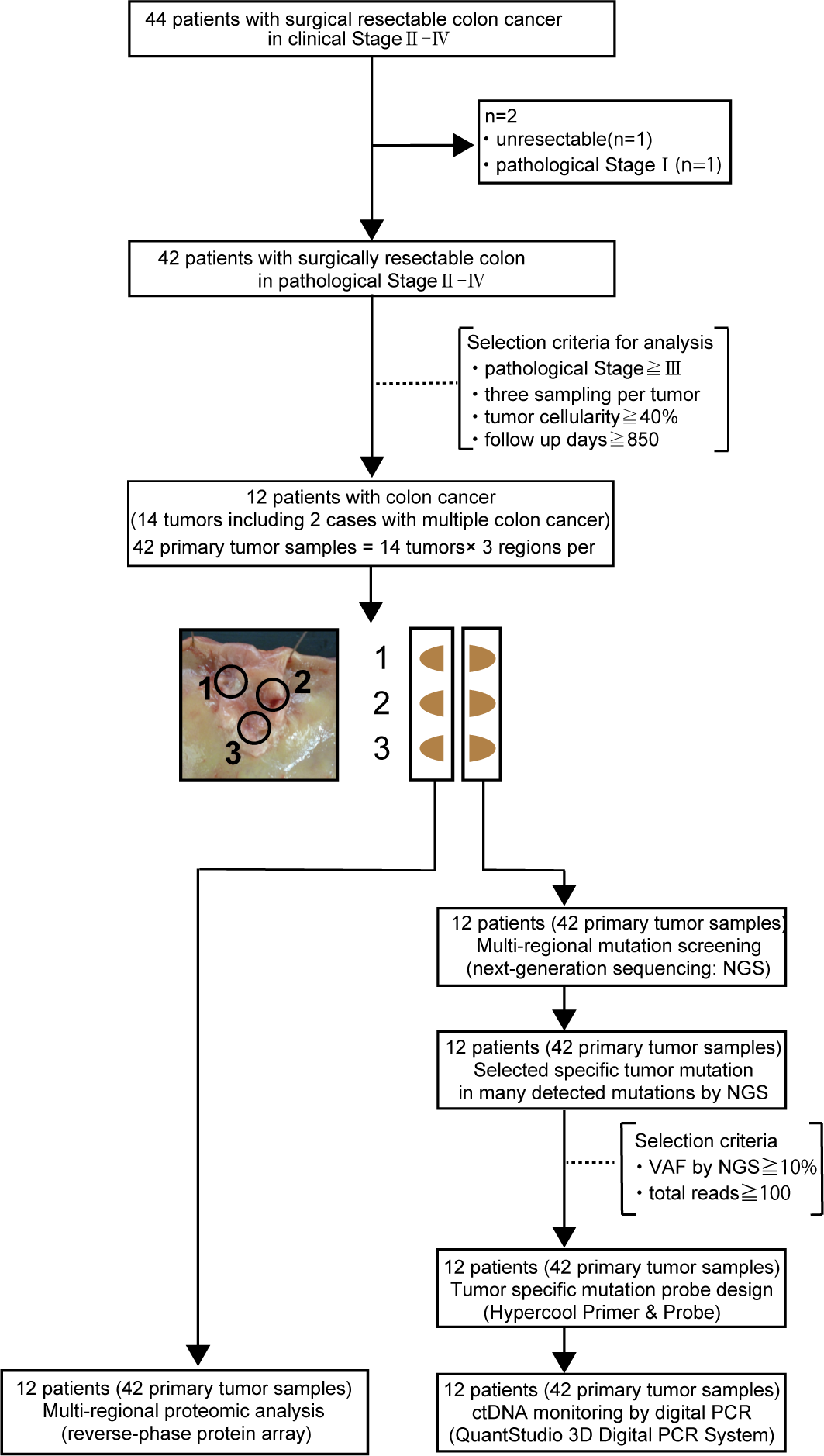


**Supplementary Figure 1**. **Flow chart of HGH28-15 study**

For current study, the subject samples were selected based on the criteria for multi-regional sequencing and proteomic study.^29^ The sequencing and proteomic data are available at the following accession numbers:

DDBJ: JGAS00000000243

MD Anderson TCPA portal: TCPA00000006-1


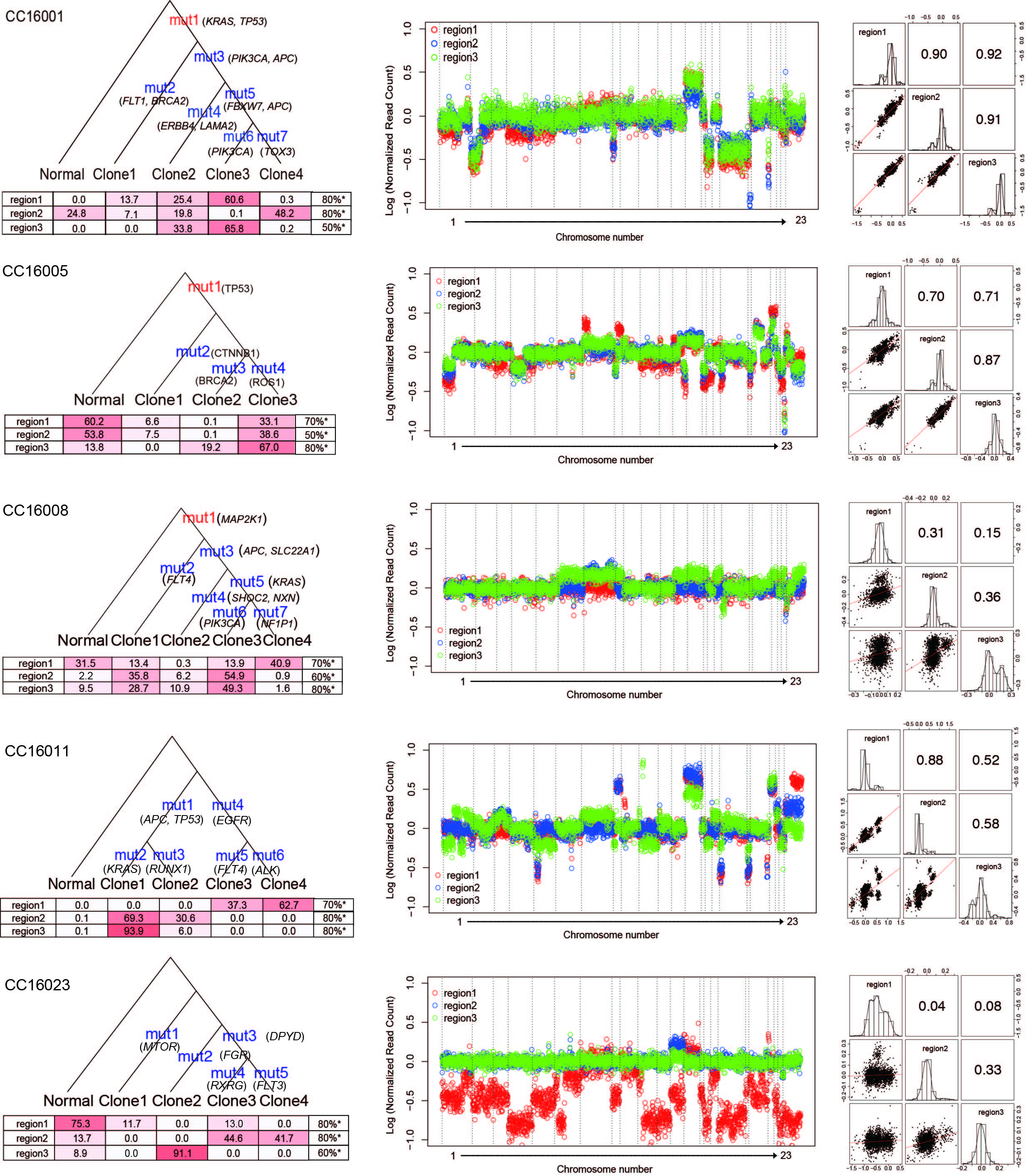


**Supplementary Figure 2**. **Intra-tumor genetic heterogeneity represented by phylogenetic tree and CNV**


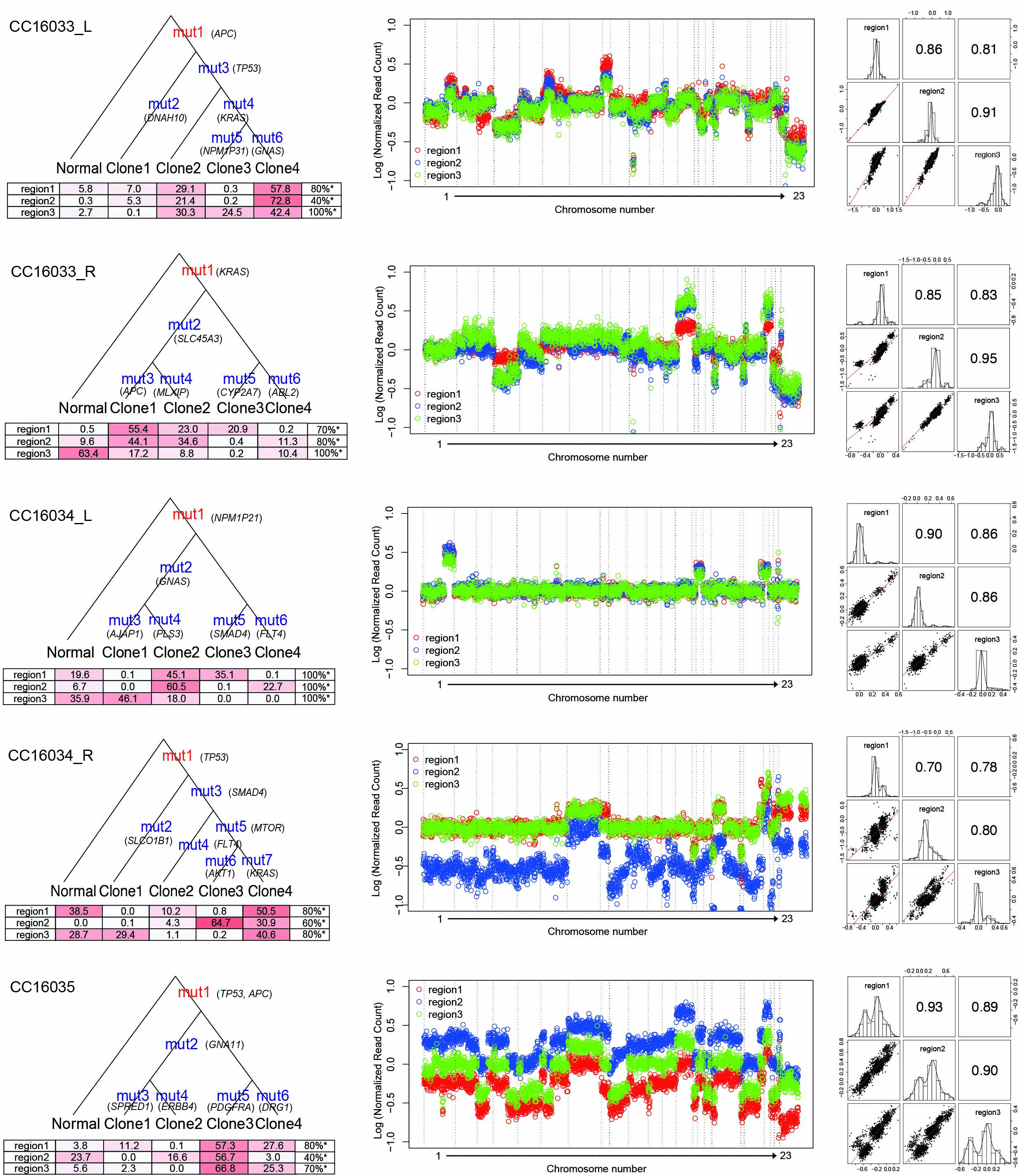


**Supplementary Figure 2**. **Intra-tumor genetic heterogeneity represented by phylogenetic tree and CNV**


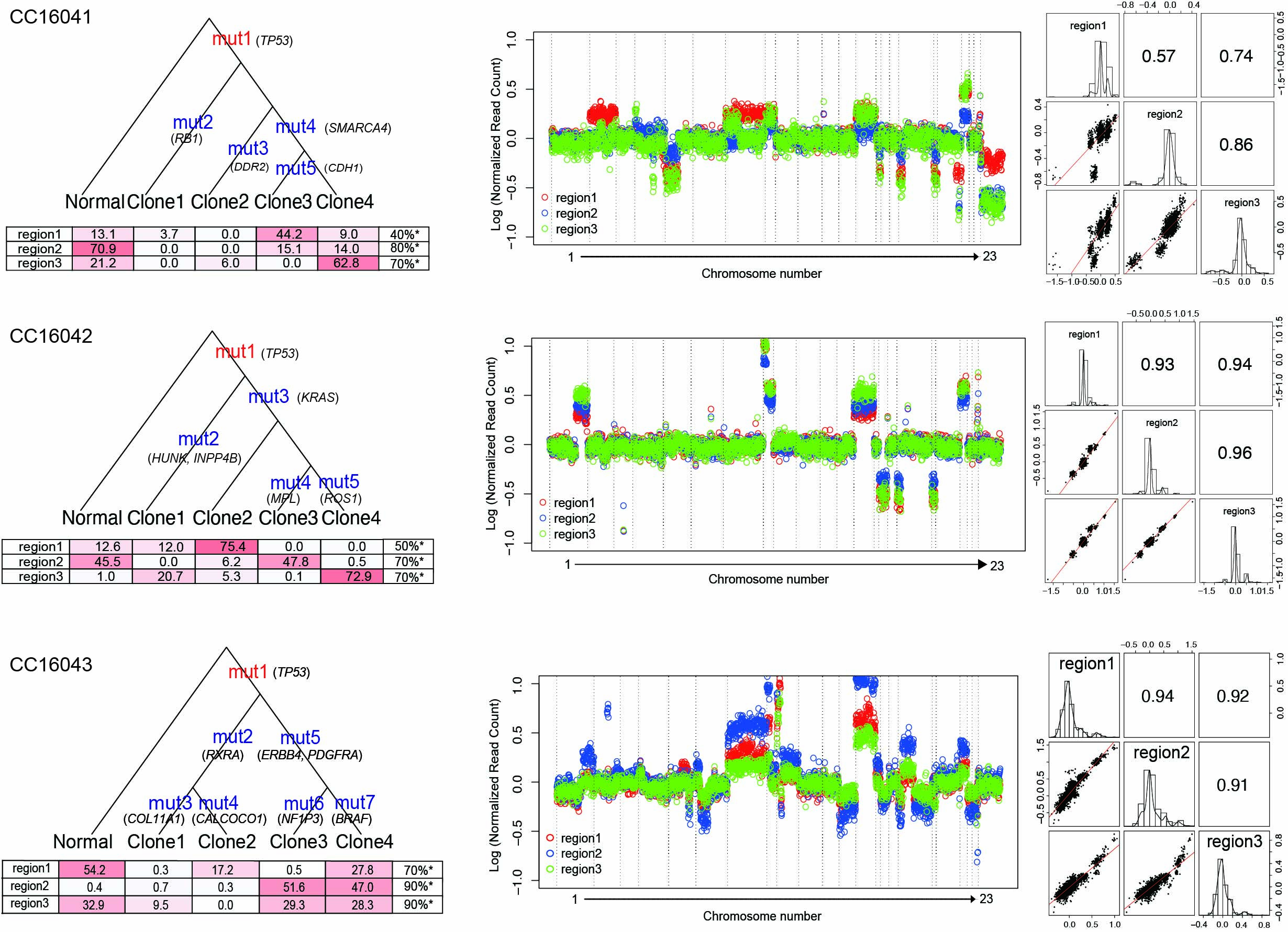


**Supplementary Figure 2**. **Intra-tumor genetic heterogeneity represented by phylogenetic tree and CNV**

Phylogenetic trees of the primary tumor are shown on the left. Truncal and branch mutations are shown in red and blue text, respectively. The proportions (%) of each clone for the three sample regions of the primary tumor are indicated at the bottom of the tree. The set of mutations per sample is presented in Data file S1. The plots in the middle panel indicate copy number across chromosomes in three regions of a tumor. Vertical axes show copy numbers represented by log transformed read counts. The right panels indicate the Pearson’s correlation coefficients for all possible combinations between the three sample regions. Scatter plots show two variables of a given pair whereas histograms show the frequency distribution within a region. ＊; Cellularity as the pathological content (%) was indicated at each region for primary tumor.

**
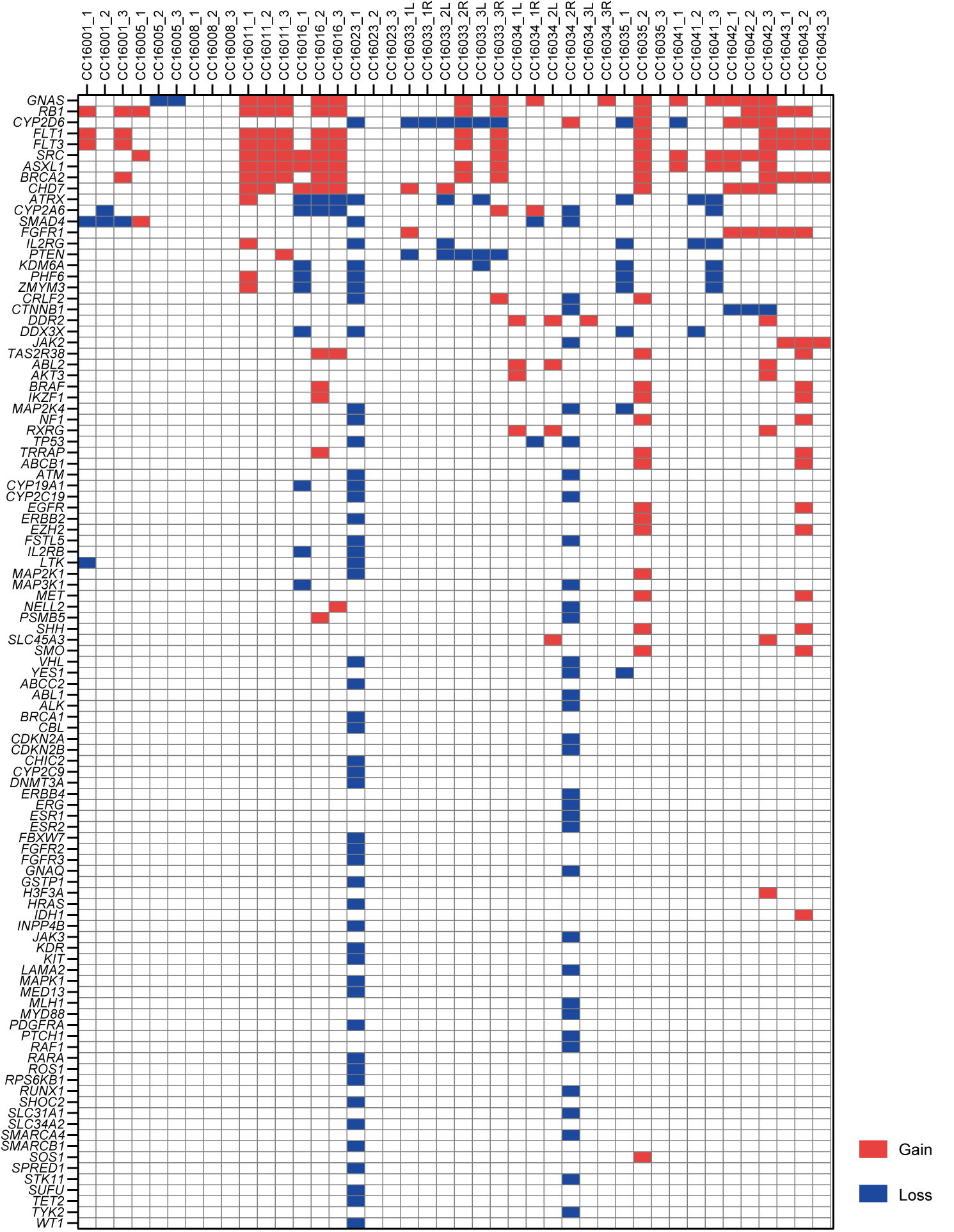
**

**Supplementary Figure 3**. **Copy number variations (CNVs) detected in each sample**

Samples are aligned by ID order at the top of the panel. Genes are indicated on the left. Red and blue squares represent CN gain and loss, respectively.


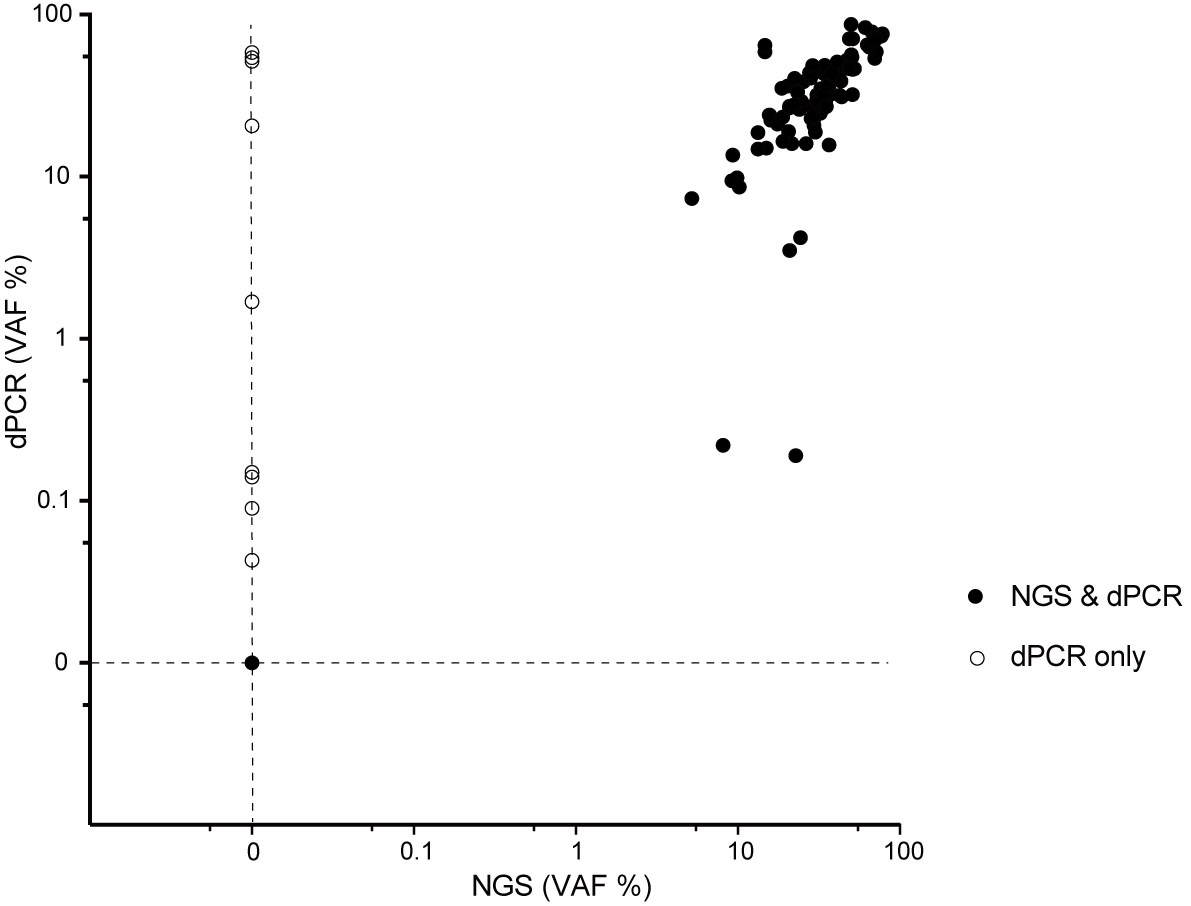


**Supplementary Figure 4**. **Correlation of NGS and dPCR findings for VAFs in primary tumors**

DNA samples for VAF comparison were extracted from primary tumor tissues. The same DNA were used in both NGS and dPCR. The VAFs for 121 mutations in primary tumors were detected using both NGS and dPCR. A total of 103 mutations were detected by both NGS and dPCR and 10 were detected by only dPCR. For both NGS and dPCR, 8 mutations could not be detected.


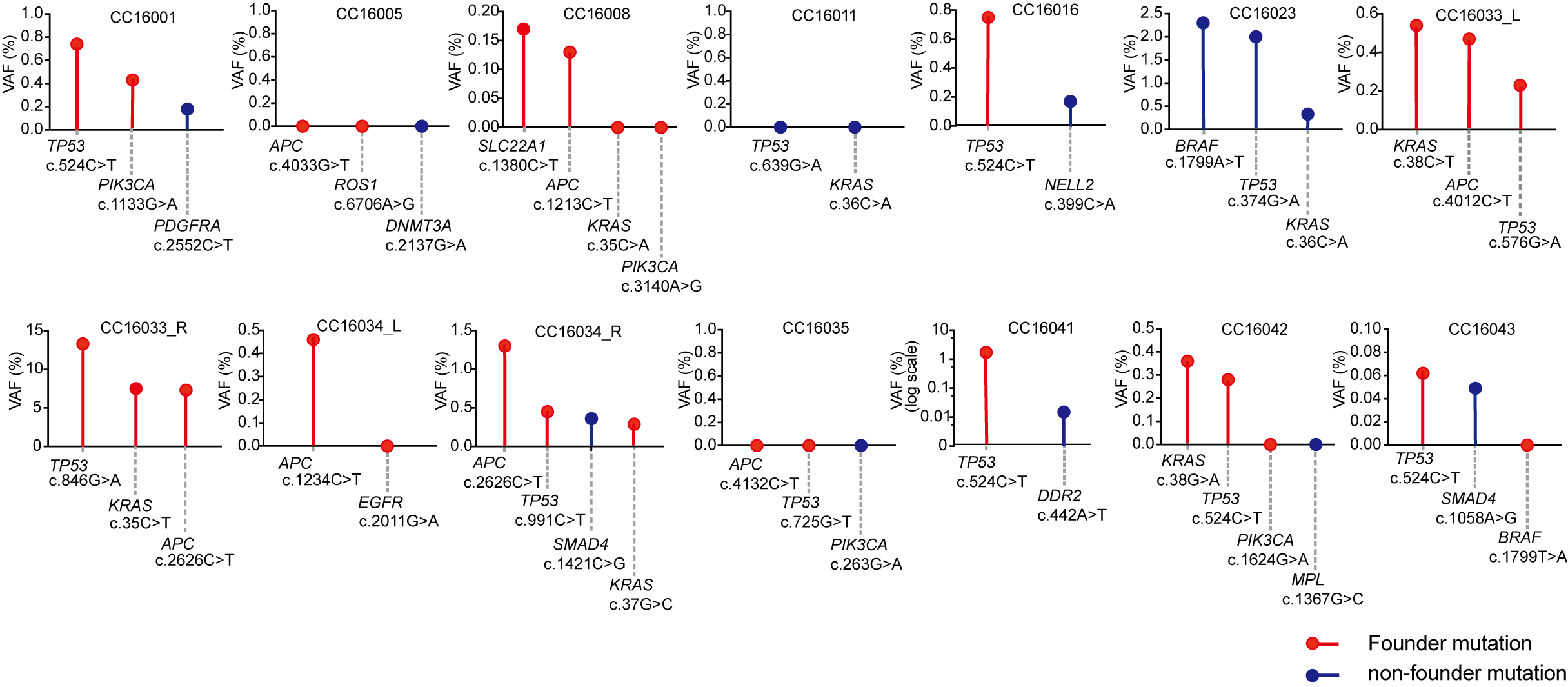


**Supplementary Figure 5** **VAFs of ctDNAs by founder and non-founder mutation**

VAFs of ctDNA are based on preoperative plasma samples. Red and blue lines show founder and non-founder mutations, respectively.


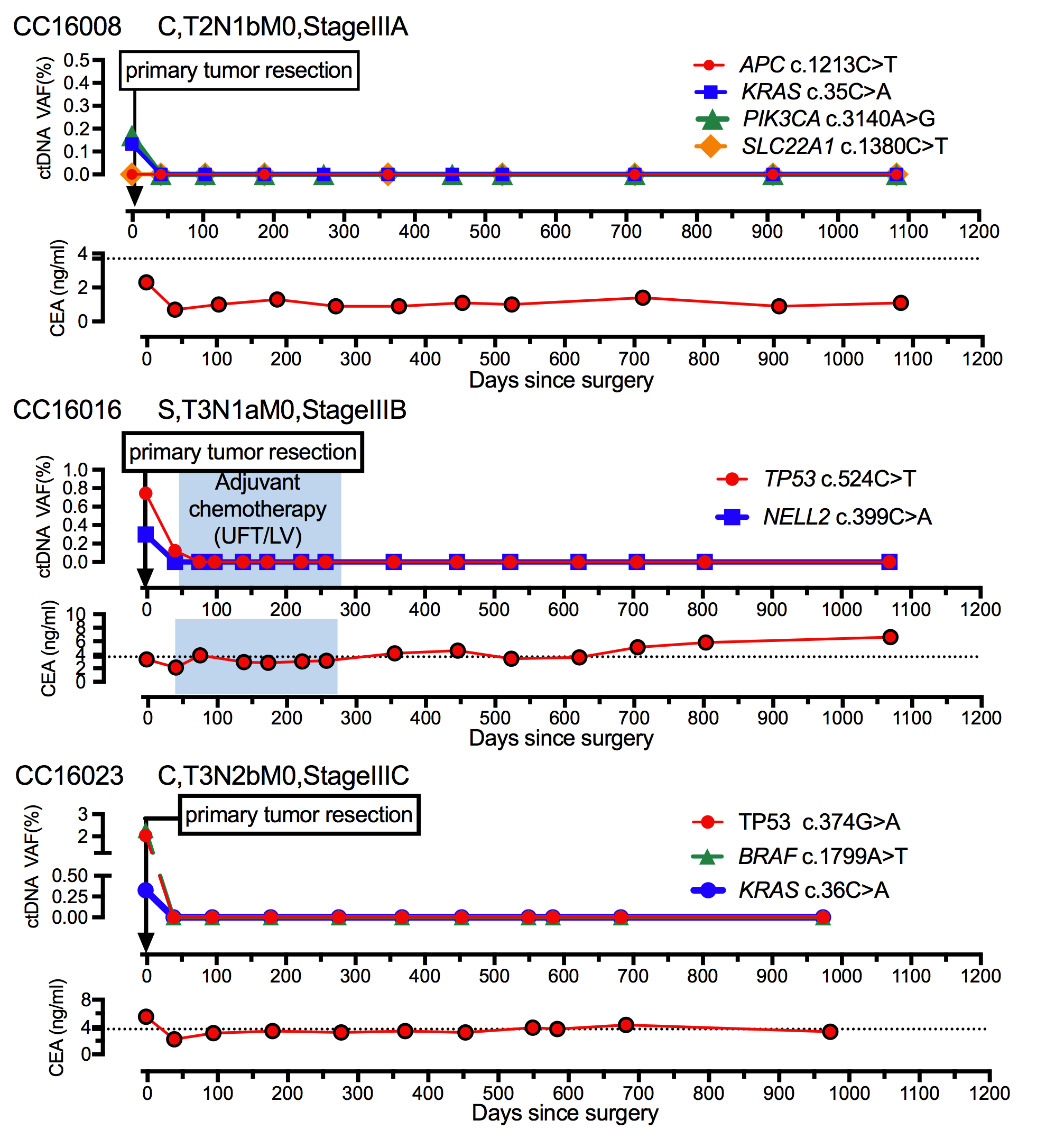


**Supplementary Figure 6**. **No recurrence corroboration is seen with ctDNA monitoring (continued)**

**
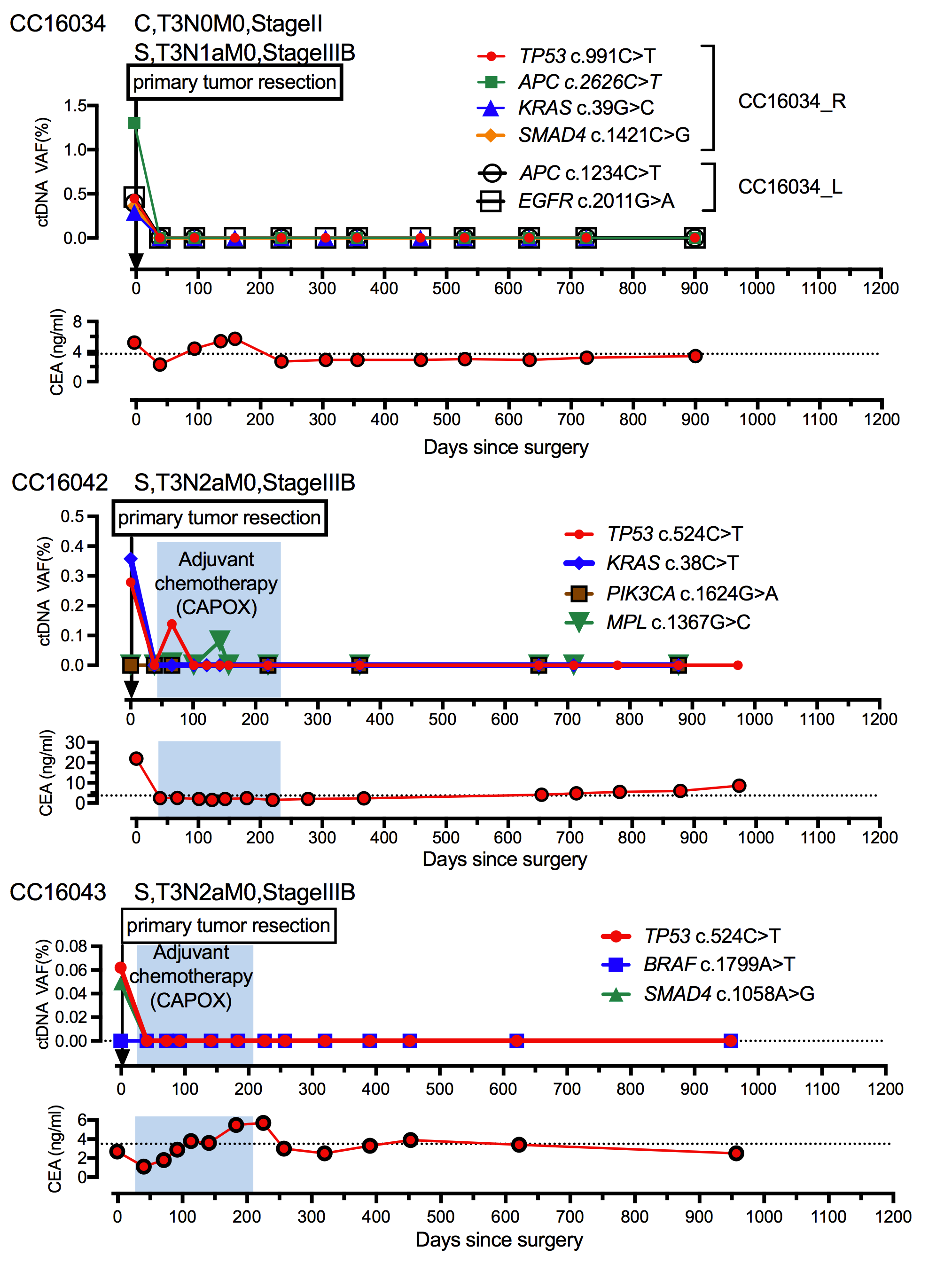
**

**Supplementary Figure 6**. **No recurrence corroboration is seen with ctDNA monitoring**

Tumor burden monitoring time course of cases with clinically non-recurrent findings is shown. Despite monitoring with multiple ctDNAs using dPCR, the ctDNA levels dropped after primary tumor resection and remained undetectable.


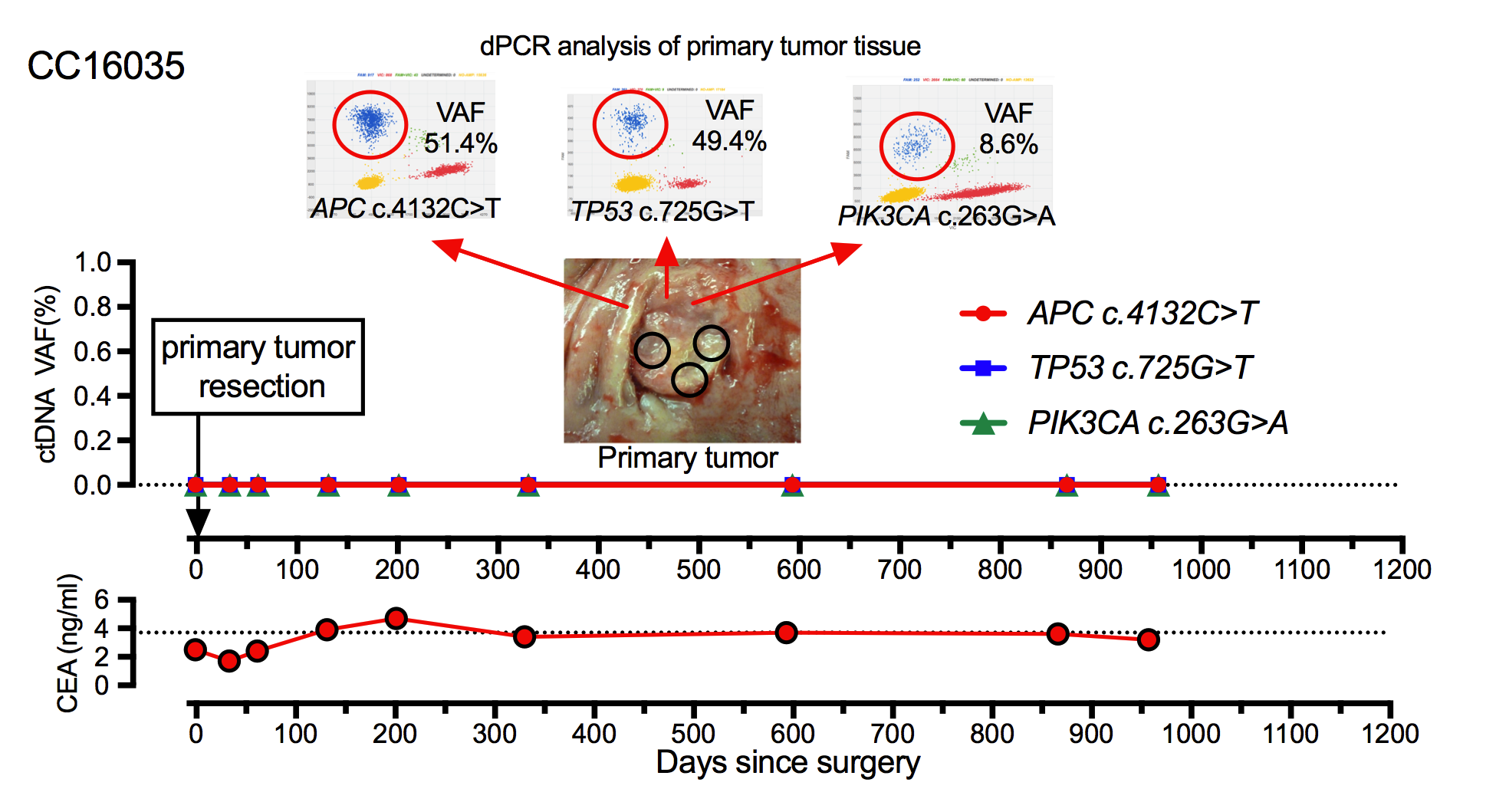


**Supplementary Figure 7**. **A** **case having undetectable ctDNA throughout the clinical course**

This case did not have recurrence and the three mutations as ctDNA were not detectable, but were detected in tumor tissue by dPCR.


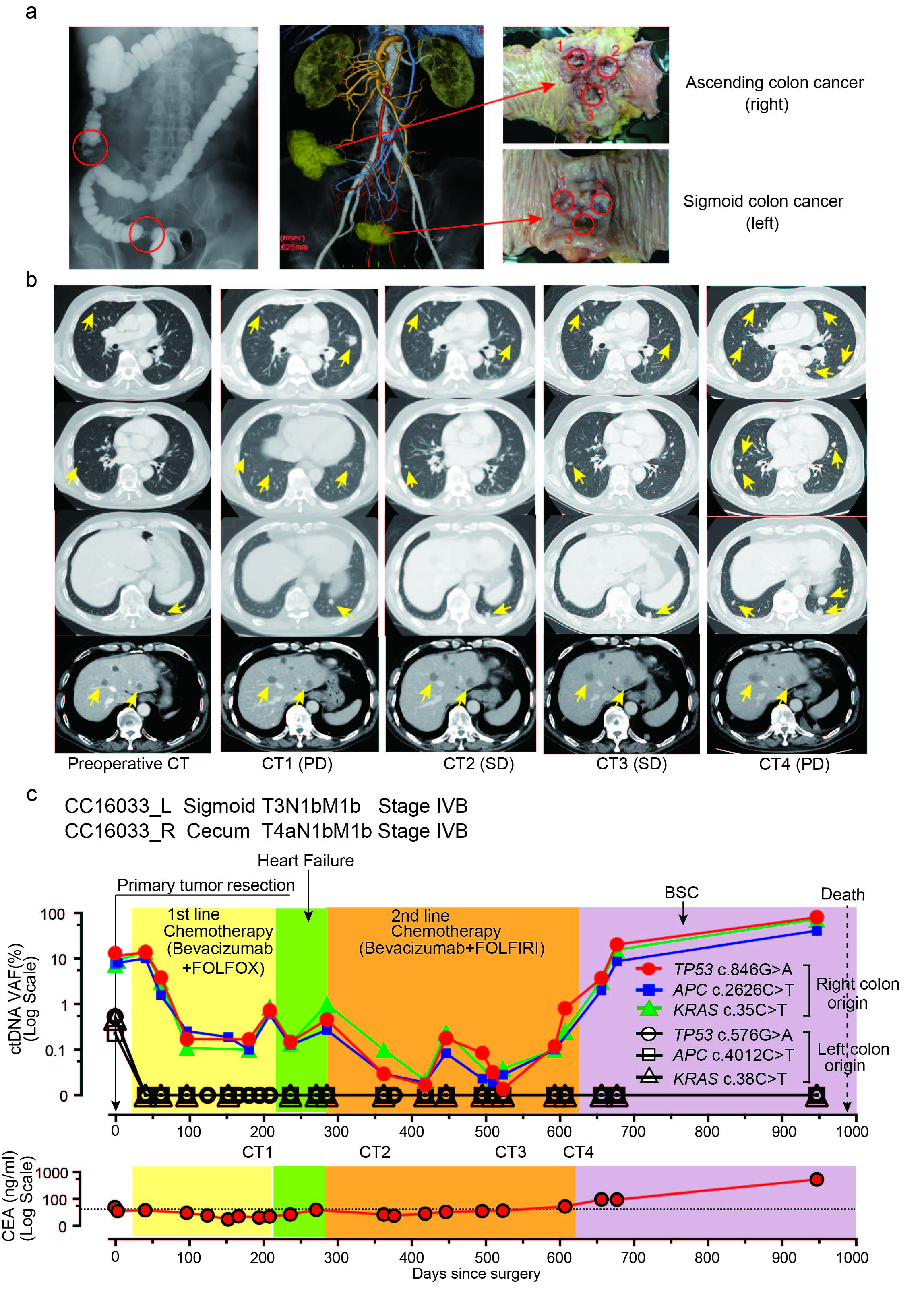


**Supplementary Figure 8. Information of a patient with multiple cancer and synchronous metastases**

(a) A macroscopic abdominal view of a patient having two tumors in the cecum (right) and sigmoid colon (left). Multi-regional samples were taken from each tumor. (b) CT imaging studies of the pre- and post-operative course for synchronous metastases to liver and lung. Yellow arrows indicate metastatic lesions. (c) A set of three mutations identified from the right colon tumor were detected as ctDNA after resection of both primary tumors and reflected the effect of chemotherapy. Another set of three mutations identified from the left colon tumor was only detected in pre-operative plasma as ctDNA and subsequently remained undetectable. PD, progressive disease. SD, stable disease. BSC, best supportive care.

**Supplementary Table 1**. **Targeted disease-associated genes in cancer panel (n=151)**

| *ABL1* | *BRCA1* | *EGFR* | *JAK2* | *MYC* | *PIK3CA* | *RUNX1* |
| --- | --- | --- | --- | --- | --- | --- |
| *AKT1* | *BRCA2* | *ESR1* | *KRAS* | *MYD88* | *PTCH1* | *SMO* |
| *ALK* | *CDKN2A* | *FGFR2* | *KIT* | *NF1* | *PTEN* | *STK11* |
| *APC* | *CEBPA* | *FLT3* | *MAP2K2* | *NOTCH1* | *PTPN11* | *TET2* |
| *ASXL1* | *CTNNB1* | *HRAS* | *MET* | *NPM1* | *NRAS* | *TP53* |
| *ATM* | *DNMT3A* | *IDH1* | *MLL* | *MTOR* | *RB1* | *VHL* |
| *BRAF* | *ERBB2* | *IDH2* | *MPL* | *PDGFRA* | *RET* | *WT1* |
| *ABCB1* | *CYP19A1* | *FBXW7* | *IL2RB* | *MLH1* | *ROS1* | *SMARCB1* |
| *ABCC2* | *CYP2A6* | *FGFR1* | *IL2RG* | *MST1R* | *RPS6KB1* | *SNCAIP* |
| *ABL2* | *CYP2B6* | *FGFR3* | *INPP4B* | *NELL2* | *RXRA* | *SOS1* |
| *AKT2* | *CYP2C19* | *FGFR4* | *JAK1* | *PDGFRB* | *RXRB* | *SPRED1* |
| *AKT3* | *CYP2C9* | *FLT1* | *JAK3* | *PHF6* | *RXRG* | *SRC* |
| *ATRX* | *CYP2D6* | *FLT4* | *KDM6A* | *PIK3R1* | *SHH* | *SUFU* |
| *CBL* | *DDR1* | *FSTL5* | *KDR* | *PSMB1* | *SHOC2* | *TAS2R38* |
| *CDA* | *DDR2* | *GNA11* | *LAMA2* | *PSMB2* | *SLC22A1* | *TRRAP* |
| *CDH1* | *DDX3X* | *GNAQ* | *LCK* | *PSMB5* | *SLC22A2* | *TYK2* |
| *CDKN2B* | *DPYD* | *GNAS* | *LTK* | *PSMD1* | *SLC31A1* | *UGT1A1* |
| *CHD7* | *ERBB3* | *GSTP1* | *MAP2K1* | *PSMD2* | *SLC34A2* | *YES1* |
| *CHIC2* | *ERBB4* | *H3F3A* | *MAP2K4* | *RAF1* | *SLC45A3* | *ZMYM3* |
| *CREBBP* | *ERG* | *HNF1A* | *MAP3K1* | *RARA* | *SLCO1B1* |  |
| *CRLF2* | *ESR2* | *IKZF1* | *MAPK1* | *RARB* | *SMAD4* |  |
| *CSF1R* | *EZH2* | *IL2RA* | *MED13* | *RARG* | *SMARCA4* |  |

**Supplementary Table 2**. **Patient information**

|  | Age | Gender | Primary tumor | Size (mm) | Pathological findings (TNM-8) | | | | | Preoperative metastasis organ |
| --- | --- | --- | --- | --- | --- | --- | --- | --- | --- | --- |
|  |  |  |  |  | T | depth | N | M | pStage |  |
| CC16001 | 72 | F | Cecum | 47×60 | 4a | SE | 1a | 0 | IIIB | − |
| CC16005 | 73 | F | Rectsigmoid | 22×20 | 2 | MP | 1b | 0 | IIIA | − |
| CC16008 | 72 | F | Cecum | 26×20 | 2 | MP | 1b | 0 | IIIA | − |
| CC16011 | 53 | F | Sigmoid | 37×48 | 4a | SE | 1a | 1a | IVA | Aorta LN |
| CC16016 | 53 | M | Sigmoid | 28×28 | 3 | SS | 1a | 0 | IIIB | − |
| CC16023 | 84 | F | Cecum | 55×82 | 3 | SS | 2b | 0 | IIIC | − |
| CC16035 | 63 | M | Rectum | 21×17 | 3 | SS | 2a | 0 | IIIB | − |
| CC16041 | 67 | M | Rectum | 35×42 | 3 | SS | 2a | 0 | IIIB | − |
| CC16042 | 69 | F | Sigmoid | 54×33 | 3 | SS | 2a | 0 | IIIB | − |
| CC16043 | 59 | F | Transverse | 47×28 | 3 | SS | 2a | 0 | IIIB | − |
| CC16033_L | 65 | M | Sigmoid | 30×25 | 3 | SS | 1b | 1b | IVB | multiple lung  &  multiple liver |
| CC16033_R | 65 | M | Cecum | 65×85 | 4a | SE | 1b | 1b | IVB |  |
| CC16034_L | 80 | F | Sigmoid | 43×60 | 3 | SS | 1a | 0 | IIIB | − |
| CC16034_R | 80 | F | Cecum | 48×65 | 3 | SS | 0 | 0 | IIA |  |

LN, lymph node; MP, muscularis propria; SE, serosa; SS: subserosa

**Supplementary Table 3.** **Mutations in primary tumors**

|  | Founder mutation | Non-founder mutation | *p* value |
| --- | --- | --- | --- |
| Average number of mutations per tumor [range] | 3.7 [0 - 9] | 8.4 [0 - 55] | 0.24^†^ |
| Total regions detected | 153 | 147 | - |
| Variant allele frequency (%) | 30.0 [22.9 – 41.4] * | 22.4 [12.1 – 38.8] * | < 0.0001^†^ |

*median [interquartile range]. ^†^Mann-Whitney’s U test.

**Supplementary Table 4**. **Comparison between truncal mutation and branch mutations**

| Case (n, Truncal : Branch) | Truncal mutation | Branch mutation | *p* value |
| --- | --- | --- | --- |
| CC16001 (6 : 47) | 71.5 [55.7-80.5] | 26.7 [20.1-37.1] | < 0.0001^†^ |
| CC16005 (3 : 22) | 27.3 [20.8-50.4] | 14.6 [6.1-19.32] | 0.0095 |
| CC16008 (3 : 29) | 54.1 [26.5-63.0] | 25.5 [11.6-29.6] | 0.0005 |
| CC16016 (6 : 24) | 59.1 [49.0-67.9] | 13.5 [6.9-22.1] | < 0.0001 |
| CC16033_L (3 : 23) | 50.2 [48.9-50.7] | 14.8 [6.6-32.6] | 0.003 |
| CC16033_R (6 : 24) | 49.4 [17.9-53.8] | 17.0[9.4-35.4] | 0.0053 |
| CC16034_L (3 : 42) | 42.9 [38.2-45.3] | 23.1 [10.8-30.5] | 0.0014 |
| CC16034_R (8 : 53) | 38.6 [35.6-57.4] | 23.3 [15.8-34.6] | 0.0009 |
| CC16035 (6 : 25) | 46.6 [40.1-48.9] | 8.4 [6.6-13.8] | < 0.0001 |
| CC16041 (6 : 29) | 41.8 [13.1-49.0] | 15.5 [9.3-30.1] | 0.0228 |
| CC16042 (3 : 39) | 62.9 [36.0-76.7] | 28.3 [13.4-36.4] | < 0.0001 |
| CC16043 (3 : 38) | 36.7 [20.3-75.0] | 14.3 [8.9-23.2] | 0.0008 |

VAF (%), median[IQR]. VAF: variant allele frequency. IQR; interquartile range. ^†^Student’s *t*-test.

**Supplementary Table 5**. **Tumor-specific mutations in each primary tumor**

|  | | Mutation | VAF (%) of mutation by NGS | | | Founder  or  Non-Founder | mut | Truncal or Branch | Preoperative plasma | | cfDNA  (ng） |  |
| --- | --- | --- | --- | --- | --- | --- | --- | --- | --- | --- | --- | --- |
|  |  |  | Tumor region | | |  |  |  | ctDNA detection | VAF (%) of ctDNA |  |  |
|  |  |  | 1 | 2 | 3 |  |  |  |  |  |  |  |
| CC16001 | | *TP53* c.524C>T | 78.0 | 43.9 | 64.9 | Founder | mut1 | Truncal | + | 0.74 | 3.2 | missense_variant |
|  |  | *PIK3CA* c.1133G>A | 43.8 | 25.4 | 39.4 | Founder | mut4 | Branch | + | 0.43 |  | missense_variant |
|  |  | *PDGFRA* c.2552C>T | 37.1 | − | 20.9 | non-Founder | mut6 | Branch | + | 0.18 |  | missense_variant |
| CC16005 | | *APC* c.1213C>T | 30.8 | 17.6 | 15.0 | Founder | mut5 | Branch | − | 0.0 | 5.4 | stop_gained |
|  |  | *ROS1* c.6708T>C | 34.9 | 20.6 | 17.7 | Founder | mut5 | Branch | − | 0.0 |  | missense_variant |
|  |  | *DNMT3A* c.2137G>A | 30.8 | 18.9 | 15.7 | non-Founder | mut5 | Branch | − | 0.0 |  | synonymous_variant |
| CC16008 | | *KRAS* c.35C>A | 21.6 | 29.5 | 27.7 | Founder | mut2 | Branch | + | 0.13 | 5.9 | missense_variant |
|  |  | *APC* c.1213C>T | 29.6 | 30.1 | 20.8 | Founder | mut2 | Branch | − | 0.0 |  | stop_gained |
|  |  | *SLC22A1* c.1380C>T | 31.4 | 32.3 | 33.4 | Founder | mut2 | Branch | − | 0.0 |  | synonymous_variant |
|  |  | *PIK3CA* c.3140A>G | 26.4 | 28.3 | 8.1 | Founder | mut4 | Branch | + | 0.17 |  | missense_variant |
| CC16011 | | *TP53* c.639G>A | 71.7 | 65.0 | − | non-Founder | mut4 | Truncal | − | 0.0 | 5.4 | stop_gained |
|  |  | *KRAS* c.36C>A | 34.4 | 22.4 | − | non-Founder | mut5 | Branch | − | 0.0 |  | missense_variant |
| CC16016 | | *TP53* c.524C>T | 67.2 | 54.0 | 70.0 | Founder | mut1 | Truncal | + | 0.75 | 4.3 | missense_variant |
|  |  | *NELL2* c.399C>A | 9.3 | − | − | non-Founder | mut6 | Branch | + | 0.17 |  | missense_variant |
| CC16023 | *KRAS* c.36C>A | | 51.2 | − | − | non-Founder | mut1 | Branch | + | 0.33 | 6.2 | missense_variant |
|  | *TP53* c.374G>A | | − | 24.7 | 5.2 | non-Founder | mut4 | Branch | + | 2.0 |  | missense_variant |
|  | *BRAF* c.1799A>T | | − | 20.8 | − | non-Founder | mut6 | Branch | + | 2.3 |  | missense_variant |
| CC16033_L | *TP53* c.576G>A | | 52.5 | 45.3 | 42.1 | Founder | mut1 | Truncal | + | 0.23 | 1.1 | stop_gained |
|  | *APC* c.4012C>T | | 50.7 | 50.2 | 48.9 | Founder | mut3 | Branch | + | 0.47 |  | stop_gained |
|  | *KRAS* c.38C>T | | 32.6 | 40.7 | 32.5 | Founder | mut3 | Branch | + | 0.54 |  | missense_variant |
|  |  | |  |  |  |  |  |  |  |  |  |  |
| CC16033_R | *TP53* c.576G>A | | 19.0 | 51.2 | 61.3 | Founder | mut1 | Truncal | + | 13.3 | 1.1 | missense_variant |
|  | *KRAS* c.35C>T | | 14.7 | 48.8 | 50.0 | Founder | mut3 | Branch | + | 7.3 |  | missense_variant |
|  | *APC* c.2626C>T | | 13.3 | 41.0 | 38.0 | Founder | mut5 | Branch | + | 7.5 |  | stop_gained |
| CC16034_L | *EGFR* c.2011G>A | | 33.6 | 35.2 | 23.4 | Founder | mut1 | Truncal | + | 0.46 | 1.7 | missense_variant |
|  | *APC* c.1234C>T | | 33.4 | 29.4 | 22.8 | Founder | mut1 | Truncal | − | 0.0 |  | stop_gained |
| CC16034_R | *TP5*3 c.991C>T | | 29.0 | 71.5 | 35.2 | Founder | mut1 | Truncal | + | 0.45 | 1.7 | synonymous_variant |
|  | *APC* c.2626C>T | | 23.4 | 42.6 | 22.1 | Founder | mut5 | Branch | + | 1.3 |  | stop_gained |
|  | *SMAD4* c.1421C>G | | − | 39.8 | 36.6 | non-Founder | mut5 | Branch | + | 0.36 |  | stop_gained |
|  | *KRAS* c.39G>C | | 18.7 | 15.6 | 24.4 | Founder | mut7 | Branch | + | 0.29 |  | missense_variant |
| CC16035 | *APC* c.4132C>T | | 48.8 | 36.7 | 46.3 | Founder | mut1 | Truncal | − | 0.0 | 3.2 | stop_gained |
|  | *TP53* c.725G>T | | 46.9 | 41.3 | 49.2 | Founder | mut1 | Truncal | − | 0.0 |  | missense_variant |
|  | *PIK3CA* c.263G>A | | − | 10.2 | − | non-Founder | mut3 | Branch | − | 0.0 |  | missense_variant |
| CC16041 | *TP53* c.524C>T | | 43.3 | 14.7 | 51.0 | Founder | mut1 | Truncal | + | 1.7 | 14.8 | missense_variant |
|  | *DDR2* c.442A>T | | 9.2 | − | − | non-Founder | mut3 | Branch | + | 0.015 |  | missense_variant |
| CC16042 | *TP53* c.524C>T | | 76.7 | 36.0 | 62.9 | Founder | mut1 | Truncal | + | 0.28 | 2.4 | missense_variant |
|  | *KRAS* c.38C>T | | 33.9 | 24.0 | 38.1 | Founder | mut4 | Branch | + | 0.36 |  | missense_variant |
|  | *PIK3CA* c.1624G>A | | 38.5 | 34.6 | 28.1 | Founder | mut4 | Branch | − | 0.0 |  | missense_variant |
|  | *MPL* c.1367G>C | | − | 28.3 | – | non-Founder | mut5 | Branch | − | 0.0 |  | missense_variant |
| CC16043 | *TP53* c.524C>T | | 36.7 | 75.0 | 20.3 | Founder | mut1 | Truncal | + | 0.062 | 17.5 | missense_variant |
|  | *BRAF* c.1799A>T | | 13.3 | 25.5 | 16.0 | Founder | mut3 | Branch | − | 0.0 |  | missense_variant |
|  | *SMAD4* c.1058A>G | | − | − | 9.9 | non-Founder | mut7 | Branch | + | 0.049 |  | missense_variant |
